# Supplementary material for: Fine Structure and Optical Features of the Compound Eyes of Adult Female Ceratosolen gravelyi (Hymenoptera: Agaonidae)
Source: Insects. 2025 Jun 30;16(7):682. doi: 10.3390/insects16070682 (PMC12295297; doi:10.3390/insects16070682)

Figure S1. A slightly oblique longitudinal section through the fused rhabdomeres reveals that two pairs of opposing neighboring retinula cells exhibit orthogonal microvilli alignment: one pair aligned uniformly in a single direction, and the other pair parallel to each other but perpendicular to the first. CC, cone cell; MT, mitochondria; PGP, pigment granule of primary pigment cell; PPC, primary pigment cell.

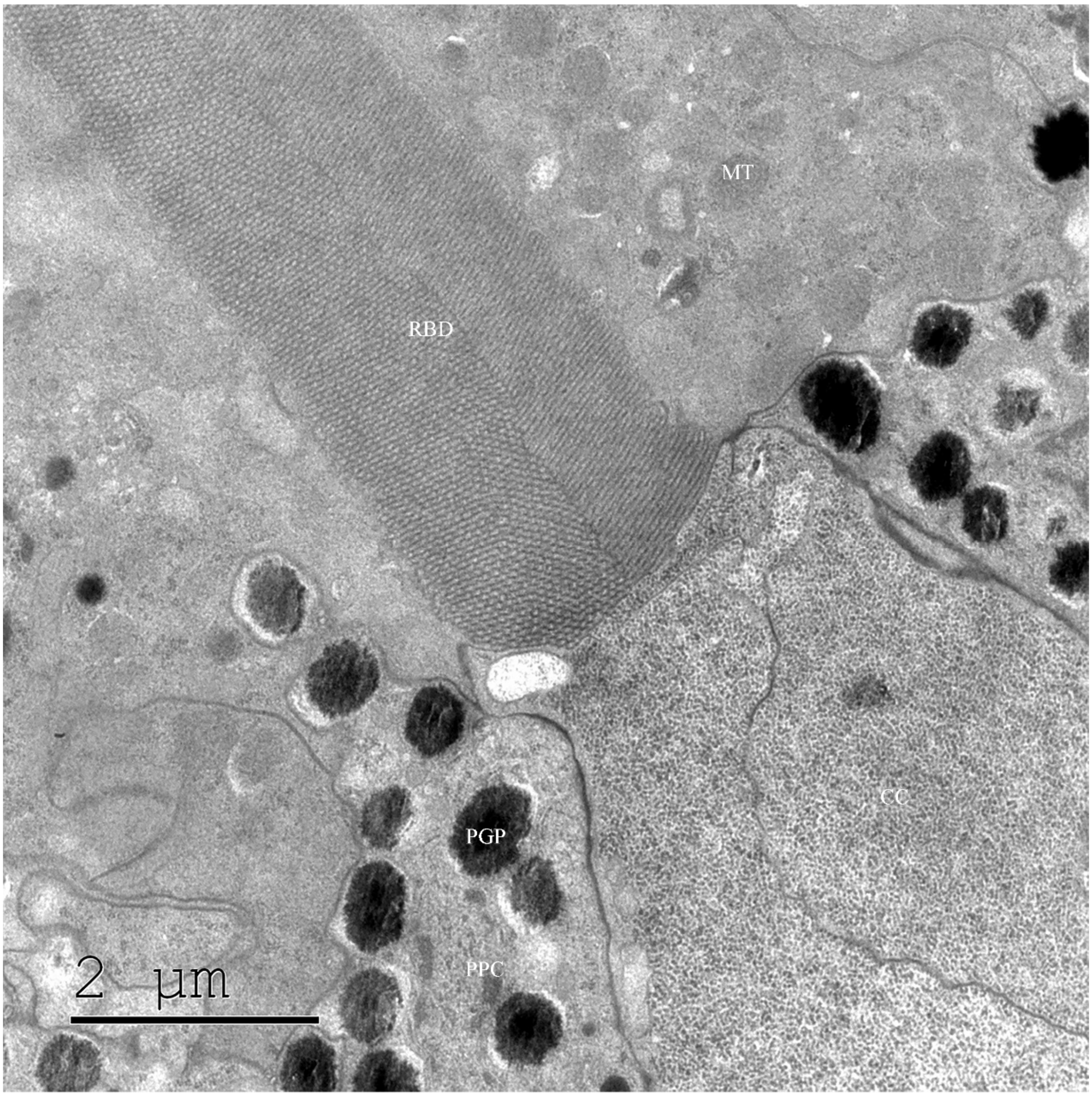

Supplement: Supplementary file 1 [file insects-16-00682-s001.zip › Supplementary Figure S1.pdf]
